# PGC-1α alleviates mitochondrial dysfunction via TFEB-mediated autophagy in cisplatin-induced acute kidney injury

**DOI:** 10.18632/aging.202653

**Published:** 2021-03-10

**Authors:** Longhui Yuan, Yujia Yuan, Fei Liu, Lan Li, Jingping Liu, Younan Chen, Jingqiu Cheng, Yanrong Lu

**Affiliations:** 1 Key Laboratory of Transplant Engineering and Immunology, NHFPC, Department of Nephrology, Frontiers Science Center for Disease-Related Molecular Network, West China Hospital, Sichuan University, Chengdu, China

**Keywords:** PGC-1α, TFEB, autophagy, acute kidney injury, mitochondrial dysfunction

## Abstract

Because of the key role of impaired mitochondria in the progression of acute kidney injury (AKI), it is striking that peroxisome proliferator γ coactivator 1-α (PGC-1α), a transcriptional coactivator of genes involved in mitochondrial biogenesis and autophagy, protects from kidney injury. However, the specific mechanism involved in PGC-1α-mediated autophagy remains elusive. *In vivo*, along with the severe kidney damage, the expression of PGC-1α was decreased in cisplatin-induced AKI mice. Conversely, PGC-1α activator (ZLN005) administration could alleviate kidney injury. Consistently, *in vitro* overexpression of PGC-1α or ZLN005 treatment inhibited cell apoptosis and mitochondrial dysfunction induced by cisplatin. Moreover, ZLN005 treatment increased the expression of LC3-II and co-localization between LC3 and mitochondria, suggesting that the mitophagy was activated. Furthermore, PGC-1α-mediated the activation of mitophagy was reliant on the increased expression of TFEB, and the protective effects were abrogated in TFEB-knockdown cells. These data suggest that the activation of PGC-1α could alleviate mitochondrial dysfunction and kidney injury in AKI mice via TFEB-mediated autophagy.

## INTRODUCTION

Acute kidney injury (AKI), defined as a rapid loss of renal function resulting from various etiologies, is a serious disease with unacceptable morbidity and mortality that continues to be a global public health concern impacting more than 13 million patients per year [1, 2]. Cisplatin (Cisp) is a platinum-based chemotherapeutic drug that has been used worldwide to treat various types of cancer, such as breast, metastatic ovarian, testicular, colorectal, lung, and neuroblastoma [3, 4]. However, the clinical use of Cisp is frequently limited by severe side-effects to normal tissues, and the most clinically significant and common toxicity is nephrotoxicity [5]. Approximately one-third of patients treated with Cisp develop renal dysfunction. Therefore, Cisp-induced AKI is considered as one of the major side-effects of Cisp therapy [6].

The mechanism of Cisp nephrotoxicity has been the focus of intense investigation for many years. Uptake of Cisp is mainly through the organic transporter pathway. The kidney accumulates cisplatin to a greater degree than other organs and is the major route for its excretion. The Cisp concentration in proximal tubular epithelial cells is approximately 5 times that of the serum concentration, which leads to subsequent tubular cell death and AKI [7–9]. To our knowledge, the proximal tubular epithelial cells are characterized by mitochondrial enrichment and a high energy consumption that is required to meet the tremendous energy demands of tubular reabsorption and secretion, and they are particularly vulnerable to various insults, such as hypoxia, oxidative stress, and toxins [10]. Extensive studies have demonstrated that mitochondria are an important target organelle in Cisp-induced AKI [11–13]. Due to accumulation in the mitochondria of renal proximal tubular cells, Cisp evokes the subsequent excessive production of reactive oxygen species (ROS), decreasing membrane potential and impairing the mitochondrial redox balance, and ultimately leading to cisplatin-induced renal injury [14–16]. Therefore, mitochondria potentially act as a target for novel therapeutic intervention to alleviate tubular injury in AKI.

Autophagy is a dynamic process that mediates the degradation of cytoplasmic components, such as damaged mitochondria, other compromised organelles, and protein aggregates, through lysosome-mediated degradation to promote cellular homeostasis [17]. An increasing number of studies have revealed that the basal autophagy in the kidney is vital for the normal homeostasis of the proximal tubules [18]. Deletion or downregulation of key autophagy genes impairs renal function and increases the p62 level and oxidative stress [19, 20]. Dong and colleagues found that autophagy activation could protect renal tubular epithelial cells by reducing mitochondrial damage and ROS production in renal ischemia-reperfusion injury [21]. Moreover, the latest research has demonstrated that conditional deleted autophagy-related genes caused more apoptosis, mitochondrial dysfunction, and tissue damage in the Cisp-induced AKI models [22]. Thus, autophagy plays an important role in improving renal function and mitochondrial damage in AKI.

Peroxisome proliferator-activated receptor γ coactivator-1 alpha (PGC-1α), a master of mitochondrial biogenesis, has been shown to be proactive in AKI. Reduction of PGC-1α has been presented in the development of diabetic kidney disease and renal fibrosis [23, 24]. PGC-1α deficiency in the proximal tubule was shown to worsen tubular injury and renal dysfunction in Cisp-induced AKI [25]. However, several strategies for enhancing the expression of PGC-1α could protect renal tubular cells from many stressors via improving mitochondrial dysfunction [23]. Despite the regulatory role of PGC-1α on mitochondrial biogenesis, recent research has found that PGC-1α could activate autophagy. For example, in vascular smooth muscle cells, PGC-1α deficiency impairs lysosomal function and autophagic flux [26]. More importantly, Tsunemi’s laboratory revealed that PGC-1α rescues Huntington’s disease proteotoxicity by promoting the transactivation of TFEB, a master regulator of the autophagy-lysosome pathway [27], suggesting that PGC-1α may activate autophagy through a TFEB-dependent mechanism [28, 29]. In this study, we investigated whether up-regulation of PGC-1α could alleviate Cisp-induced AKI, and whether the renal protective effects rely on TFEB-mediated autophagy.

## RESULTS

### Expression of PGC-1α was down-regulated in AKI mice

**Figure 1:**
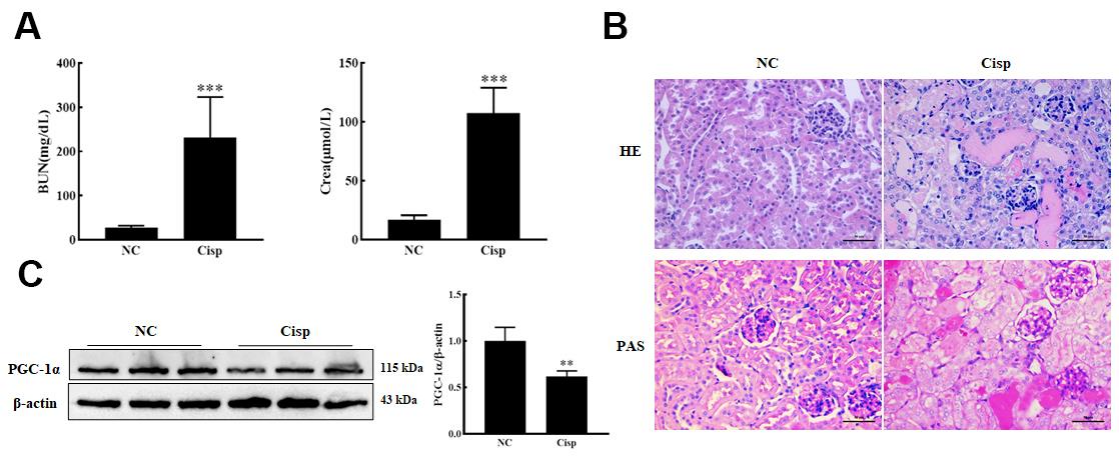
**The expression of PGC-1α was down-regulated in AKI mice.** The male C57BL/6 mice were injected with a single dose of cisplatin (16mg/kg, i.p.) to induce AKI and sacrificed on 4th day. (**A**) Serum BUN and Crea levels were quantified in each group (n=10). (**B**) Representative images of HE- and PAS-stained kidney sections. Scar bar: 50 μm. (**C**) Representative images of western blotting and the quantitative analysis of PGC-1α. Data are provided as the mean ± SEM, n=3 independent experiments. **P < 0.01, ***P < 0.001 vs. Con (NC, normal control; Cisp, cisplatin; BUN, blood urea nitrogen; Crea, serum creatinine; HE, hematoxylin and eosin; PAS, periodic acid-Schiff.).

To evaluate the variation of PGC-1α in cisplatin-induced AKI mice, male C57BL/6 mice were intraperitoneally injected with Cisp (16 mg/kg) to induce AKI and sacrificed on the 4th day. Levels of blood urea nitrogen (BUN) and creatinine (Crea) were significantly increased in Cisp-injected mice compared to the control mice (Figure 1A). PAS and HE staining of the kidney sections revealed that Cisp-treated mice displayed histopathological alterations such as marked edema, vacuolar degeneration and the narrowing of tubular lumen (Figure 1B). As is well known, Cisp can induce the apoptosis of proximal tubular epithelial cells. It was reported that the depletion of PGC-1α in proximal tubular epithelial cells significantly aggravated the severity of tubular injury in Cisp-induced AKI, indicating that PGC-1α may play an important role in AKI. Similar to previous studies, the expression of PGC-1α in the AKI group was decreased compared with that in the NC group (Figure 1C). Taken together, these results demonstrate that PGC-1α is repressed in Cisp-induced AKI.

### PGC-1α alleviates Cisp-induced injury in HK2 cells

**Figure 2:**
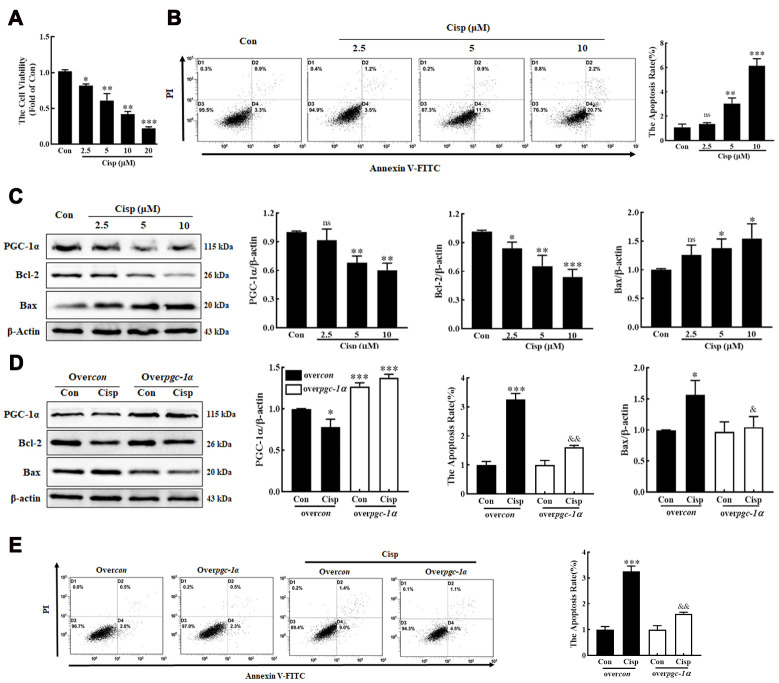
**PGC-1α alleviates cisplatin-induced injury in HK2 cells.** HK2 cells were exposed to cisplatin (2.5 μM, 5 μM, 10 μM) for 48h. (**A**) Cell viability was determined by CCK8 assay. (**B**) Cisplatin-induced cell apoptosis were determined by flow cytometry. (**C**) The expression of apoptosis-related proteins (Bax and Bcl-2) and PGC-1α was measured by western blotting. (**D**) Over-PGC-1α attenuated cell apoptosis in cisplatin-treated (5 μM) HK2 cells (over*con*, black column; over*pgc-1α*, white column). The expression of PGC-1α and Bcl-2 and Bax was analyzed by western blotting. (**E**) Apoptosis was determined by flow cytometry. Data are provided as the mean ± SEM, n=3 independent experiments. *P < 0.05, **P < 0.01, ***P < 0.001 vs. Con; ^&^P < 0.05, ^&&^P < 0.01 vs. Cisp. (over*con*, overcontrol; Cisp, cisplatin).

**Figure 3:**
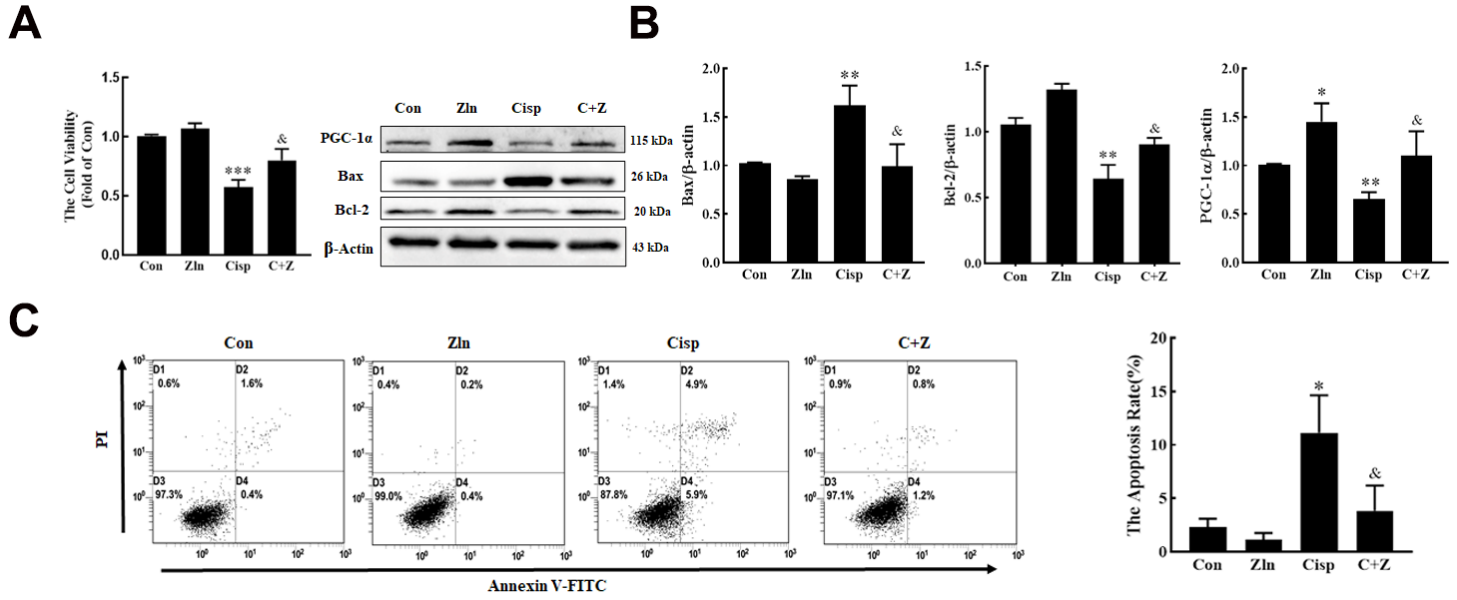
**Pharmacological activation of PGC-1α by ZLN005 treatment suppresses cisplatin-induced injury *in vitro*.** HK2 cells treated with cisplatin (5 μM) were incubated with ZLN005 (10 μM) for 48 h. (**A**) cell viability was determined by CCK8 assay. (**B**) The expression of apoptosis-related proteins (Bax and Bcl-2) and PGC-1α was measured by western blotting. (**C**) The effects of ZLN005 on cisplatin-induced apoptosis were determined by flow cytometry. Data are provided as the mean ± SEM, n=3 independent experiments. *P < 0.05, **P < 0.01, ***P < 0.001 vs. Con; ^&^P < 0.05 vs. Cisp. (Con, control; Zln, ZLN005; Cisp, cisplatin; C+Z, cisplatin + ZLN005).

To determine whether PGC-1α plays a protective role in Cisp-treated HK2 cells, Cisp-treated HK2 cells were incubated with Zln (ZLN005, an activator of PGC-1α, 10 μM) or transfected with the pgc1α-Flag-His plasmid to activate PGC-1α. We found that after Cisp treatment for 48h, cell viability was decreased in a concentration-dependent manner (Figure 2A). In addition, cell apoptosis was increased after Cisp treatment (Figure 2B). Meanwhile, the protein level of Bax was increased after Cisp treatment, while the protein levels of PGC-1α and Bcl-2 were decreased (Figure 2C). After transfection with the pgc-1α-Flag-His plasmid prior to Cisp treatment, the cell apoptosis induced by Cisp was significantly inhibited (Figure 2D). In parallel to inhibition of cell apoptosis, the enhanced expression of Bax was decreased; conversely, the reduced expression of Bcl-2 was increased (Figure 2E). Moreover, as shown in Figure 3A, compared with the Cisp-treated cells, pharmacological activation of PGC-1α by Zln treatment, increased cell viability from 0.53±0.029 to 0.80±0.056. Similarly, cell apoptosis induced by Cisp was inhibited after Zln treatment (Figure 3B). Meanwhile, Zln elevated the expression levels of PGC-1α and Bcl-2, inversely inhibited the expression level of Bax (Figure 3C). These data show that the activation of PGC-1α alleviates Cisp-induced injury in HK2 cells.

### Oral administration of Zln attenuates cisplatin-induced AKI

**Figure 4:**
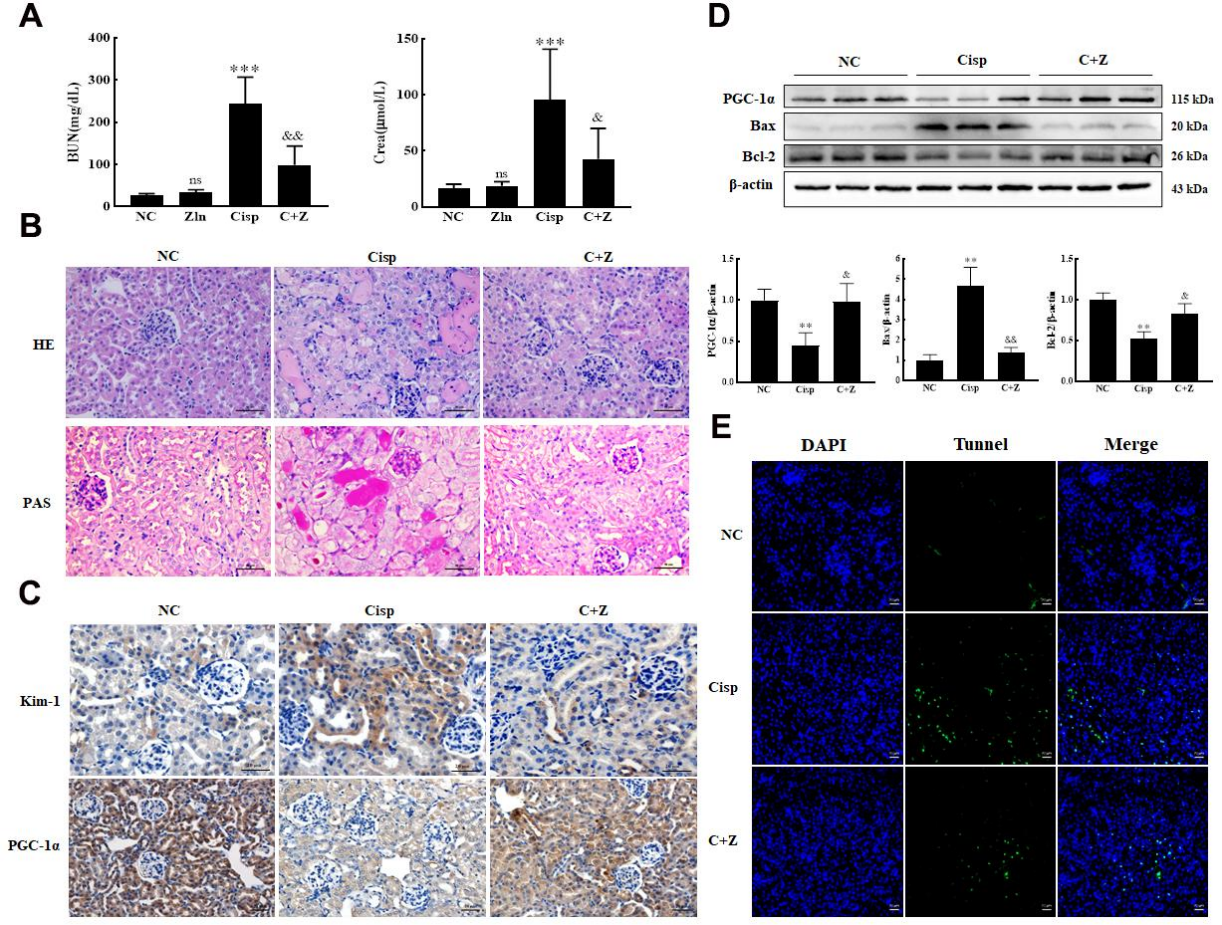
**Oral administration of ZLN005 prevents cisplatin-induced AKI.** The male C57BL/6 mice were injected once with cisplatin (16mg/kg, i.p.) to induce AKI, followed by ZLN005 treatment (15mg/kg/d, i.g.) for 4 days. (**A**) Serum BUN and Crea levels were quantified in each group (n=10). (**B**) Representative images of HE- and PAS-stained kidney sections. (**C**) Representative images of immunohistochemical staining using anti-Kim-1 and anti-PGC-1α antibodies. Scar bar: 20 μm. (**D**) Representative images of western blotting and the quantitative analysis of PGC-1α, Bax and Bcl-2. (**E**) Representative micrographs showing TUNEL staining. Data are provided as the mean ± SEM, n=3 independent experiments. *P < 0.05, **P < 0.01 vs. Con; ^&^P < 0.05, ^&&^P < 0.01 vs. Cisp. (NC, normal control; Cisp, cisplatin; C+Z, cisplatin + ZLN005).

To further explore whether Zln treatment could protect against Cisp-induced kidney injury, mice were administered Cisp (16 mg/kg, a single i.p. injection) to induce AKI, then were treated with Zln (15 mg/kg per day, i.g.) for 4 days at the beginning of AKI. We found that Zln treatment for 4 days had no effects on BUN and Crea levels, suggesting that Zln did not induce nephrotoxicity in mice (Figure 4A). The levels of BUN and Crea were increased on the 4th day after Cisp injection, but decreased after Zln administration (Figure 4A). In addition, histological staining of the kidney sections showed that the renal proximal tubular histopathological alterations induced by Cisp were attenuated after Zln administration (Figure 4B). Immunohistochemical staining revealed that the administration of Zln elevated the level of PGC-1α and reduced the level of Kim-1, a sensitive renal tubular injury marker, compared with the AKI mice (Figure 4C). Consistently, the decreased protein level of PGC-1α after Cisp injection was markedly increased by Zln (Figure 4D).

It is well known that Cisp induces the apoptosis of proximal tubular epithelial cells. We next assessed apoptosis in the kidney by terminal deoxynucleotidyl transferase-mediated dUTP nick end labeling (TUNEL) staining. We found that the number of TUNEL-positive cells was lower in the Zln-treated group than in the Cisp group (Figure 4E). Accordingly, the significantly higher expression of pro-apoptotic Bax treated with Cisp was decreased, while the anti-apoptotic Bcl-2 was increased after Zln administration (Figure 4D). Taken together, these findings suggested that Zln treatment repressed Cisp-induced kidney injury.

### Activation of PGC-1α via Zln inhibits Cisp-induced mitochondria damage *in vitro*

**Figure 5:**
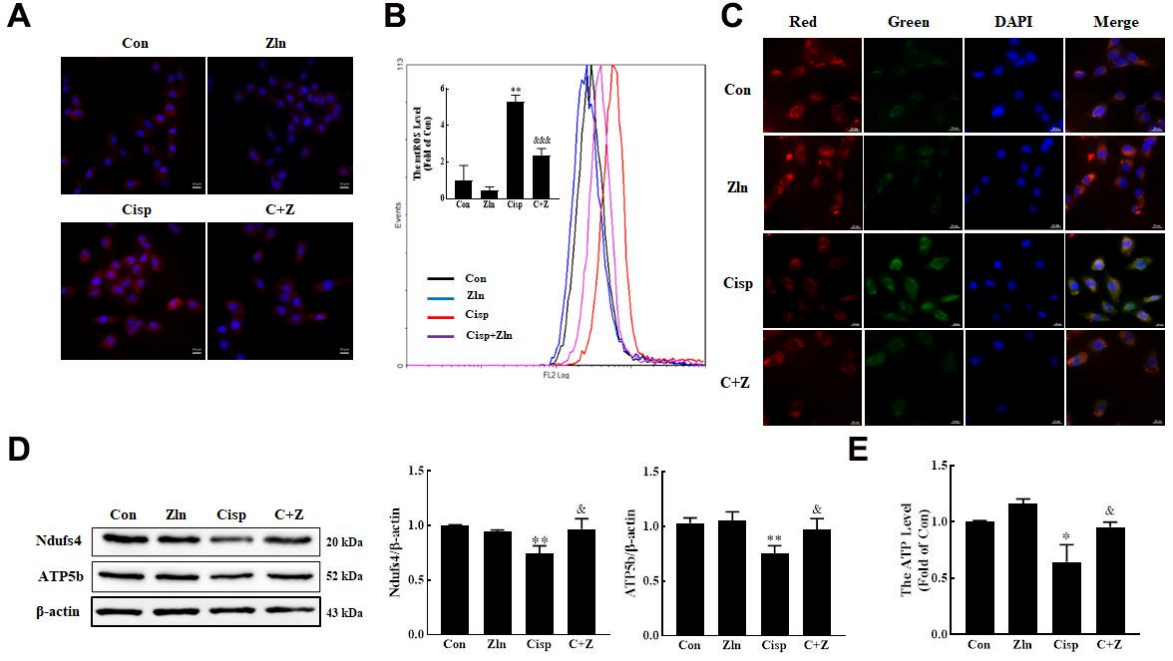
**Activation of PGC-1α via ZLN005 inhibits cisplatin-induced mitochondria damage *in vitro*.** (**A**) For the measurement of mitochondrial ROS (mtROS), HK2 cells were stained with Mito-SOX Red (2.5 μM) for 15 min at 37° C and determined by confocal microscope. Scar bar: 20 μm. (**B**) Mitochondrial ROS (mtROS) were measured by incubation with Mito-SOX Red. (**C**) The mitochondrial membrane potential measurement was detected with JC-1 (5 nM). Scar bar: 20 μm. (**D**) The expression of mitochondria-related proteins (ATP5b and Ndufs4) was measured by western blotting. (**E**) ATP content was measured by using an ATP Assay, ATP concentration was calculated in nmol/mg protein, and the data are represented as the rate of control. Data are provided as the mean ± SEM, n=3 independent experiments. *P < 0.05, **P < 0.01 vs. Con; ^&^P < 0.05, ^&&^P < 0.01, ^&&&^P< 0.001 vs. Cisp. (Con, control; Zln, ZLN005; Cisp, cisplatin; C+Z, cisplatin + ZLN005).

It is well known that Cisp causes the mitochondrial dysfunction in proximal tubular epithelial cells. Therefore, we detected the effects of Zln treatment on Cisp-induced mitochondrial damage. Here, we found that after Zln treatment, the overproduction of mitochondrial ROS (mtROS) and depolarization of membrane potential induced by Cisp was inhibited (Figure 5A–5C). In addition, the expression levels of mitochondrial transport chain complex proteins (ATP5b and Ndufs4) and ATP content were increased after Zln administration (Figure 5D, 5E). Furthermore, we found that compared with the control cells the number of mitochondria in Cisp-treated cells was increased (Supplementary Figure 1A). Based on previous research, the increased number of mitochondria in cells may be due to the improved mitochondrial biogenesis or decreased removal of damaged mitochondria [30]. In the present study, we have demonstrated that the mitochondrial function after cisplatin incubation, which led us to postulate that the increase of mitochondrial number in this scenario may be due to accumulation of damaged mitochondria. To address this hypothesis, we further detect the damaged mitochondria after Cisp treatment via Mito-tracker-green and Mito-tracker-deep-red staining, in which the Mito-tracker-green^hi^ Mito-tracker-deep-red^low^ mitochondria was indicated as damaged mitochondria as previous studies described [31, 32]. As shown in Supplementary Figure 1B, the damaged mitochondria rate was increased in Cisp-induced HK2 cells. Moreover, compared with Cisp-treatment group, the number of mitochondria in Cisp+Zln group was decreased (Supplementary Figure 1A). These data show that the activation of PGC-1α protected against Cisp-induced mitochondrial dysfunction.

### PGC-1α activates autophagy by modulating TFEB

**Figure 6:**
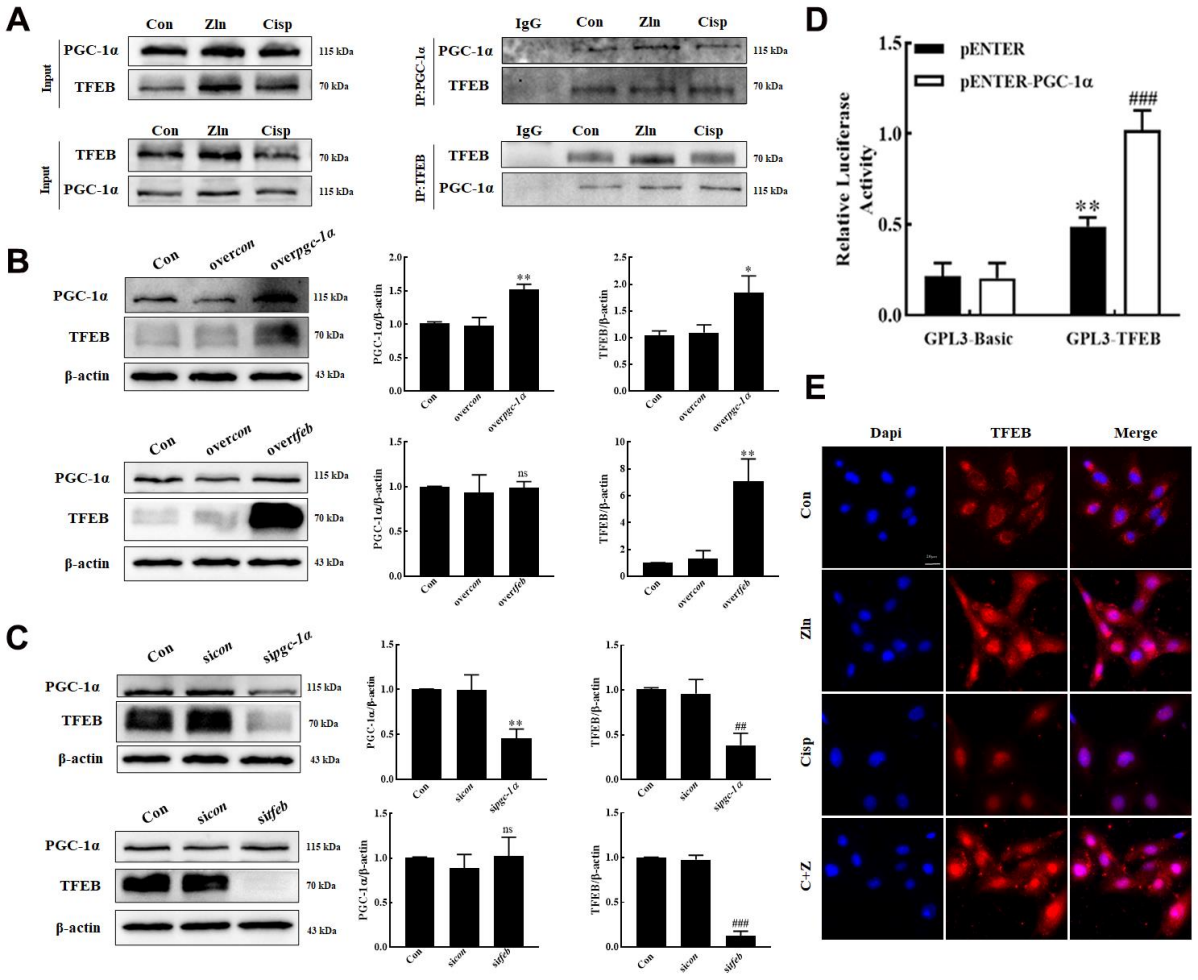
**PGC-1α positively regulate the TFEB in HK2 cells.** (**A**) HK2 Cell lysates were immunoprecipitated (IP) with an anti-PGC-1α or an anti-TFEB antibody, then immunoblotted (IB) with TFEB and PGC-1α antibodies. Anti- IgG antibody as a negative control. (**B**, **C**) The expression of TFEB and PGC-1α was measured by western blotting. (**D**) Luciferase activity in HK-2 cells transfected with the TFEB promoter-reporter construct linked to luciferase along with PGC-1α plasmids or control empty vectors. (**E**) Representative immunofluorescence images of TFEB in HK2 cells. Scar bar: 20 μm. Data are provided as the mean ± SEM, n=3 independent experiments. *P < 0.05, **P < 0.01, ***P < 0.001 vs. over*con*; ^#^P < 0.05, ^##^P < 0.01, ^###^P < 0.001 vs si*con* (Con, control; si*con*, sicontrol; over*con*, overcontrol).

Tsunemi T et al. reported that PGC-1α overexpression rescues Huntington’s disease proteotoxicity by preventing oxidative stress and promoting TFEB function [27]. To explore the interaction between PGC-1α and TFEB in HK2 cells, we performed a series of co-immunoprecipitation (Co-IP) to pull down complexes by using antibodies against PGC-1α or TFEB. Both PGC-1α and TFEB were found in the complexes, leading us to test if TFEB gene expression is regulated by PGC-1α (Figure 6A). To address the hypothesis, HK2 cells were transfected with pgc1α-Flag-His plasmid or tfeb-Flag-His plasmid. We found that along with the overexpression of PGC-1α, the expression of TFEB was increased, while no obvious change of PGC-1α was observed in TFEB-overexpressed HK2 cells (Figure 6B). Moreover, in PGC-1α-knockdown HK2 cells, the expression of TFEB was obviously decreased. However, knockdown of TFEB had no effect on PGC-1α expression (Figure 6C), noting that PGC-1α positively regulates the TFEB in HK2 cells.

To further delineate the basis of PGC-1α regulation of TFEB, we generated a TFEB promoter-reporter construct linked to luciferase and then performed transactivation assays in HK2 cells, and found that transfection of PGC-1α into HK2 cells expressing this reporter construct yielded a robust induction of luciferase activity (Figure 6D). These results indicated that PGC-1α up-regulated TFEB gene expression by directly ligating its promoter. In addition, consistent with the finding in skeletal muscle by Spaulding [33], we also observed that the nuclear localization of TFEB in HK2 cells were increased after Zln-mediated PGC-1α overexpression (Figure 6E).

**Figure 7:**
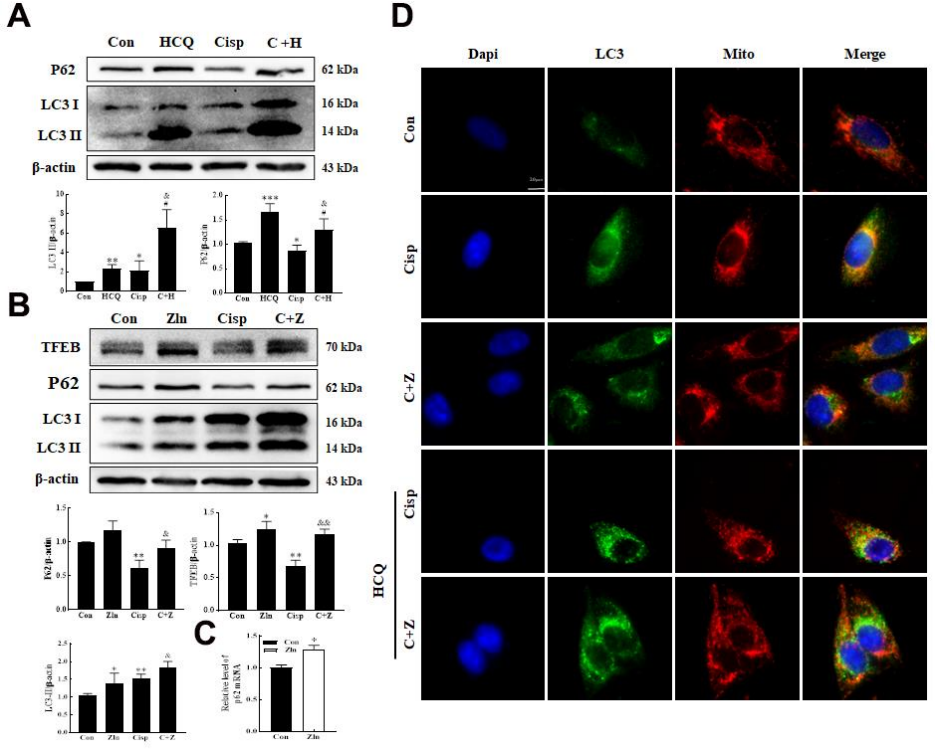
**PGC-1α activates autophagy by modulating TFEB.** (**A**) The expression of the proteins P62 and LC3 was measured by western blotting in HK2 cells exposed to cisplatin (5 μM) in the presence or absence of HCQ (30 μM) for 48 h. (**B**) HK2 cells were exposed to cisplatin in the presence or absence of ZLN005 (10 μM) for 48 h, and the expression of the proteins TFEB, P62 and LC3was measured by western blotting. (**C**) HK2 cells treated with ZLN005 (10 μM) and p62 mRNA was measured by real-time PCR. (**D**) Representative images of the colocalization between LC3 and mitochondria. Data are provided as the mean ± SEM, n=3 independent experiments. *P < 0.05, **P < 0.01 vs. Con; &P < 0.05, &&P < 0.01 vs. Cisp. (Con, control; Zln, ZLN005; Cisp, cisplatin; C+Z, cisplatin + ZLN005; HCQ, hydroxychloroquine; C+H, cisplatin + hydroxychloroquine).

Based on the crucial role of TFEB in autophagy, we hypothesized that the upregulation of PGC-1α improving mitochondrial function in Cisp-induced AKI may rely on the activation of TFEB-mediated autophagy. To test this hypothesis, we detected autophagy in Cisp-induced HK2 cells. The level of LC3 II was increased in HK2 cells treated with Cisp for 48 h, while the level of P62 was decreased (Figure 7A). According to the guidelines for the use and interpretation of assays for monitoring autophagy (3rd edition), the accumulation of LC3 II may increase from the induction of autophagy or the inhibition of autophagic degradation [34, 35]. To discriminate between these two possibilities, we treated HK2 cells with HCQ, a lysosomal acidification inhibitor. After this treatment, the LC3 II protein level was dramatically elevated in Cisp-treated HK2 cells, indicating that increased LC3 II turnover and the induction of autophagic flux were induced by Cisp in HK2 cells. The results suggested that autophagy was activated after Cisp treatment (Figure 7A). Moreover, comparing with the Cisp-treated group, protein levels of LC3 II, TFEB and P62 were increased in Cisp+Zln group (Figure 7B).

The accumulation of P62 was associated with the transcriptional activation of P62 or dysfunctional autophagy. Here, the elevated protein of P62 was due to the increase of the p62 mRNA after Zln treatment (Figure 7C). Mitophagy, as a selective autophagy, is critical for mitochondrial maintenance. Our previous research demonstrated that the expression of Pink1 and Parkin was increased after cisplatin treatment [36]. Consistently, the number of punctate LC3 structures and the co-localization between LC3 and mitochondria in Cisp-induced HK2 cells were increased after Zln treatment (Figure 7D). Moreover, compared with the Cisp+Zln group, we found that the number of punctate LC3 structures was increased in the presence of HCQ (Figure 7D). These results demonstrate that PGC-1α activates TFEB-mediated mitophagy.

**Figure 8:**
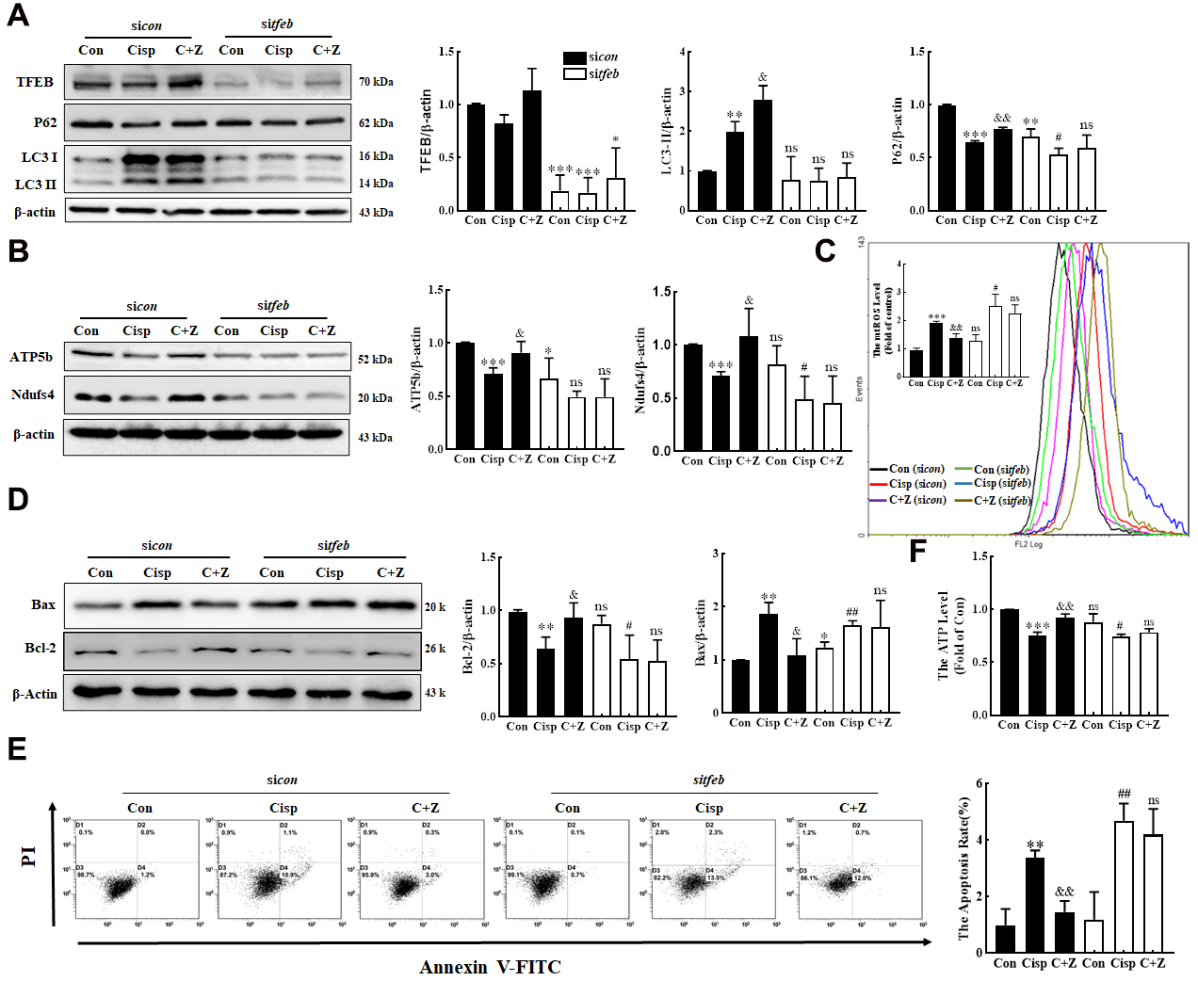
**Silencing of TFEB partially abolishes the protective effects of ZLN005 in cisplatin-treated HK2 cells.** HK2 cells were transfected with control siRNA (si*con*, black column) or TFEB siRNA (si*tfeb*, white column) for 6 h and treated with cisplatin in the presence or absence of ZLN005 for 48 h. (**A**) The expression of autophagy-related protein (TFEB, P62 and LC3) was measured by western blotting. (**B**) The expression of mitochondria-related proteins (ATP5b and Ndufs4) was measured by western blotting. (**C**) Mitochondrial ROS (mtROS) were measured by incubation with Mito-SOX Red. (**D**) The expression of apoptosis-related proteins was measured by western blotting. (**E**) The effects of ZLN005 on cisplatin-induced apoptosis were determined by flow cytometry. (**F**) ATP levels were measured by using an ATP Assay kit. Data are provided as the mean ± SEM, n=3 independent experiments. *P < 0.05, **P < 0.01, ***P < 0.001 vs. Con; ^&^P < 0.05, ^&&^P < 0.01 vs. Cisp; ^#^P < 0.05, ^##^P < 0.01 vs. si*tfeb.* (Con, control; Zln, ZLN005; Cisp, cisplatin; C+Z, cisplatin + ZLN005).

To further determine whether the protective effects of PGC-1α in Cisp-induced injury were rely on TFEB-mediated autophagy, HK2 cells were transfected with si*tfeb* prior to Zln treatment. Along with the inhibition of TFEB in TFEB-knockdown HK2 cells, the expression of LC3 II was decreased (Figure 8A). Moreover, the improved mitochondrial function and reduced cell apoptosis after Zln treatment was abrogated (Figure 8B–8F). In addition, based on the key role of TFEB in PGC-1α-mediated mitophagy, we also assess the effects of si*tfeb* on mitochondrial number, and we found that the number of mitochondria was increased comparing with the Cisp+Zln group (Supplementary Figure 2A). These results imply that PGC-1α modulates mitochondrial dysfunction via TFEB-mediated autophagy.

### Zln treatment alleviates renal injury in Cisp-induced AKI mice via PGC-1α/TFEB pathway

**Figure 9:**
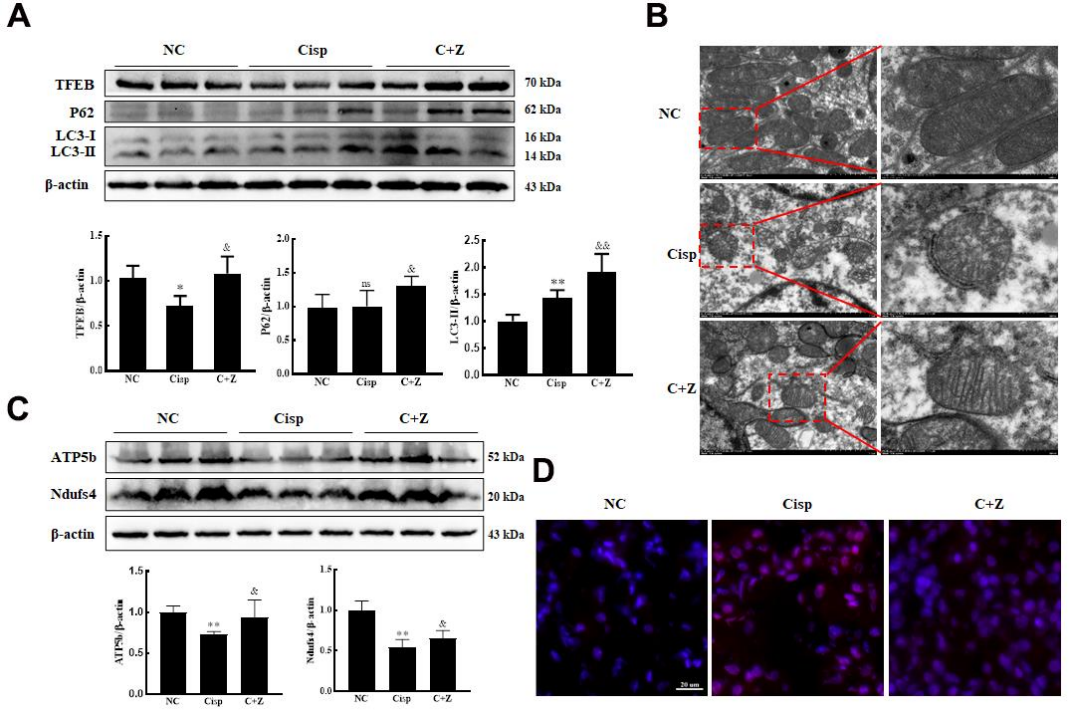
**ZLN005 treatment alleviates renal injury in cisplatin-induced AKI mice via PGC-1a/TFEB pathway.** The male C57BL/6 mice were injected once with cisplatin (16mg/kg, i.p.) to induce AKI, followed by ZLN005 treatment (15mg/kg/d, i.g.) for 4 days. (**A**) Western blots of TFEB, P62 and LC3 levels in kidney. (**B**) Representative TEM micrographs of mouse renal tubular epithelial cell mitochondria from each group. Scale bar, 2 μm (wireframe indicates the magnified image). (**C**) The expression of mitochondria-related proteins (ATP5b and Ndufs4) was measured by western blotting. (**D**) For the measurement of mitochondrial ROS (mtROS), frozen sections of freshly renal tissues were stained with Mito-SOX Red (2.5 μM) 15 min at 37° C and determined by confocal microscope. Scar bar: 20 μm. Data are provided as the mean ± SEM, n=3 independent experiments. *P < 0.05, **P < 0.01 vs. Con; ^&^P < 0.05, ^&&^P < 0.01 vs. Cisp. (NC, normal control; Cisp, cisplatin; C+Z, cisplatin + ZLN005).

Based on the previous study, Lynch/Parikh reported that at the early stage of Cisp-induced AKI the mitophagy was induced as an adaptive response, while was retarded along with the development of AKI, thereby accelerating cell death [25]. Similarly, the protein level of LC3 II in AKI mice was obviously elevated (Figure 9A). After Zln administration, in addition to the increased expression of TFEB, the expression of LC3 II in the kidney sections was furtherly increased compared with AKI mice, indicating that PGC-1α activated mitophagy in AKI mice (Figure 9A). To further investigate the effect of Zln in Cisp-induced mitochondrial damage *in vivo*, we observed the mitochondria in the cortices of mouse kidneys using transmission electron microscopy. Compared with normal control mice, Cisp-treated mice presented abnormal mitochondria with disorganized cristae, smaller and with a round appearance. However, these phenomena were obviously attenuated after Zln administration (Figure 9B). Additionally, the expression levels of mitochondrial transport chain complex proteins (ATP5b and Ndufs4) were increased and mtROS in the kidney was decreased after Zln administration compared with the Cisp-treated group (Figure 9C, 9D). Overall, these findings demonstrate that the activation of PGC-1α relieved mitochondrial dysfunction in Cisp-induced AKI mice via the PGC-1α/TFEB pathway.

## DISCUSSION

Recent studies have demonstrated a key nephroprotective role of PGC-1α in AKI models, and the effects depend on the suppression of inflammation, as well as the promotion of mitochondrial biogenesis, although the specific mechanism remains largely unclear [23, 24, 37]. Our study illustrated the mechanism involved in PGC-1α/TFEB-mediated renal protective effect in cisplatin-induced AKI mice, and the major findings are as follows: i) increasing the expression of PGC-1α via various strategies could alleviate Cisp-induced AKI; ii) PGC-1α ameliorates mitochondrial dysfunction and apoptosis via autophagy-mediated clearance of damaged mitochondria; iii) PGC-1α-activated autophagy was dependent on TFEB.

Mitochondrial dysfunction is increasingly recognized as an initiator of and contributor to AKI [15, 38]. Mitochondria undergo significant alterations during AKI, with loss of functions and damage of structure appearing earlier than the pathological manifestations of kidney injury [39, 40]. Mitochondria are the main source and target of ROS within cells [41]. Under pathological conditions, mtROS can trigger oxidative stress, the inflammatory response and apoptosis, which ultimately results in kidney injury [21, 42]. Multiple aspects of mitochondrial dysfunction, such as the overproduction of reactive oxidative species (ROS), disruption of mitochondrial membrane potential (ΔΨm), and exacerbation of apoptosis are thought to influence AKI [38]. Consistent with previous studies, we found that mitochondrial function was impaired after cisplatin treatment *in vivo* and *in vitro*, representing the excessive accumulation of mitochondrial ROS and depolarization of the membrane potential. Nonetheless, the upregulation of PGC-1α *in vivo* and *in vitro* alleviated mitochondrial dysfunction, decreased mtROS and apoptosis.

PGC-1α, as a key transcriptional regulator of the expression of mitochondrial proteins, is involved in mitochondrial biogenesis, energy homeostasis and oxidative stress [23, 24]. Recent studies have demonstrated that PGC-1α is downregulated in AKI induced by endotoxemia, nephrotoxic agents, and ischemia-reperfusion (I/R) [13, 43–46], while overexpression of PGC-1α reduces kidney injury in notch-induced kidney fibrotic mice and diabetic nephropathy mice [47, 48]. Moreover, in mouse models of AKI induced by endotoxemia and IRI, PGC-1α is suppressed following injury and returns to basal levels with recovery [37]. Similar to previous studies, the protein level of PGC-1α was reduced in AKI mice in our study, suggesting that PGC-1α may plays a critical role in renal recovery. However, the exact mechanism of the renal-protective effects of PGC-1α in AKI needs further investigation.

Mitophagy, as an evolutionarily conserved cellular process, plays an important role in maintaining the number of mitochondria and energy metabolism by clearing dysfunctional mitochondria, as well as preventing excessive ROS accumulation and apoptosis [25, 49]. Our previous research demonstrated that the expression of Parkin and Pink1 was increased after Cisp treatment, and the downregulation of mitophagy exacerbated Cisp-induced acute kidney injury [36]. Here, we revealed that the overexpression of PGC-1α induced autophagy *in vivo* and *in vitro*, as shown by the increased expression of LC3-II. Therefore, the subsequent investigation of the mechanism of PGC-1α-mediated autophagy is necessary. Transcription factor EB (TFEB) is a master regulator of the CLEAR pathway involved in autophagy and lysosomal biogenesis. Studies have demonstrated that TFEB promotes the expression of genes in the autophagy pathway, as well as genes encoding the components of lysosomes [29, 50, 51]. In the present research, we found that PGC-1α and TFEB interact with each other by Co-IP, suggesting that there is a potential link between PGC-1α and TFEB. According to previous researches, the relationship between PGC-1α and TFEB is complicated in different physiological stimuli. Several studies have proved that TFEB promotes PGC-1α transcription by directly ligating its promoter in the liver, adipocytes, and cardiomyocyte [52–54]. Moreover, in Huntington’s disease and Neuro2a cells PGC-1α places an upstream of TFEB in the transcriptional regulation of the autophagy-lysosome pathway [27]. A recent study has found that TFEB-driven lysosomal biogenesis is pivotal for PGC-1α dependent renal stress resistance, in which overexpression of PGC-1α ameliorates Cisp’s suppressive effect on TFEB [25]. Similar with the found, in our study we also observed that PGC-1α increased the expression of TFEB in HK2 cells, leading us to test if TFEB gene expression is regulated by PGC-1α. Moreover, in dual-luciferase reporter assay we found that overexpression of PGC-1α increased TFEB gene transcription by directly ligating promoter of TFEB. More importantly, TFEB knockdown remarkably abrogated the protective role of Zln in Cisp-treated HK2 cells. These findings placed PGC-1α upstream of TFEB and autophagy-lysosome pathway activation in this scenario.

In summary, this study proved that the upregulation of PGC-1α activated TFEB-mediated autophagy, resulting in alleviation of mitochondrial dysfunction and kidney injury in Cisp-induced AKI mice. These findings suggest that the activation of PGC-1α may be a potential therapeutic strategy against AKI.

## MATERIALS AND METHODS

### Antibodies and reagents

Cisplatin, ZLN005 and hydroxychloroquine (HCQ) were purchased from MedChemExpress (MCE, New Jersey, USA). The Cell Counting Kit 8 (CCK-8) assay was purchased from Dojindo (Kumamoto, Japan). The Annexin V/PI apoptosis detection kit was purchased from BD Corporation (San Jose, CA, USA). For the detection of mitochondrial ROS, mitochondrial morphology and mitochondrial membrane potential (ΔΨm), Mito-SOX Red and Mito-tracker-deep-red were obtained from Thermo Fisher Scientific (Sunnyvale, CA, USA), and a JC-1 kit was obtained from AAT Bioquest (Sunnyvale, CA). Mito-tracker-green, an ATP measurement kit and Hoechst were purchased from Beyotime Biotechnology (Jiangsu, China). The pgc-1α-Flag-His plasmid (NM_013261) and tfeb-Flag-His plasmid (CH898579) were purchased from Vigene Biosciences (Shandong, China). The tfeb-siRNA (si*tfeb*) and pgc-1α-siRNA (si*pgc-1α*) were synthesized by GenePharma (Shanghai, China). The jetPRIME transfection reagent was obtained from Polyplus Transfection (Illkirch, France). The reagents used for transmission electron microscopy (osmium tetroxide, Epon, and lead citrate) and DAPI were purchased from Sigma-Aldrich (Taufkirchen, Germany). For real-time PCR, TRIzol was purchased from Gibco (Life Technologies, CA, USA), an iScript cDNA synthesis kit was obtained from Bio-Rad (CA, USA), and the SYBR Green PCR mix was purchased from Vazyme Biotech (Nanjing, China). For western blot, radioimmuno-precipitation assay (RIPA) buffer supplemented protease inhibitor was obtained from Calbiochem (San Diego, CA, USA), and a BCA protein assay kit (Cwbio, China) was used to measure protein concentrations.

### Cell culture

HK2 cells (purchased from ATCC) were cultured in DMEM/F-12 medium (HyClone, Solarbio, Beijing, China) supplemented with 10% fetal bovine serum (FBS) and 1% antibiotics (100 U/ml penicillin and 100 μg/ml streptomycin) at 37° C in 5% CO_2_ in a humidified incubator. To detect the effect of PGC-1α on Cisp-induced cell injury, cells incubated with 5 μM Cisp for 48 h were treated with or without Zln (10 μM). To inhibit autophagy, cells were co-cultured with HCQ (30 μM).

### Cell transfection

Small interfering RNA against tfeb (si*tfeb*) (sense: GACGAAGGUUCAACAUCAATT; antisense: UUGAUGUUGAACCUUCGUCTT), si*pgc-1α* (sense: GGACAGUGAUUUCAGUAAUTT; antisense: AUUACUGAAAUCACUGUCCTT) and negative control RNAi (si*con*) (sense: UUCUCCGAACGUGUCACGUTT; antisense: ACGUGACACGUUCGGAGAATT) were purchased from GenePharma (Shanghai, China). The pgc-1α-Flag-His plasmid (NM_013261) and tfeb-Flag-His plasmid (CH898579) were purchased from Vigene Biosciences (Shandong, China). To induce the overexpression of PGC-1α or the knockdown of PGC-1α, HK2 cells were transfected with tfeb-Flag-His plasmid and si*tfeb* using the jetPRIME transfection reagent. The pgc1α-Flag-His plasmid and si*pgc-1a* were transfected into HK2 cells to induce the expression or knockdown of PGC-1α with the jetPRIME transfection reagent.

### Cell viability

Cells were seeded in a 96-well plate overnight at a density of 4 × 10^3^ /well. After treatment with Cisp (2.5 μM, 5 μM, 10 μM) or in the presence of Zln (10 μM) and incubation for 48 h, cells were washed three times with PBS and cell viability was measured using a Cell Counting Kit-8. Briefly, 10 μl of CCK-8 solution was added to each well containing 100 μl of medium for 2 hours at 37° C. The absorbance was detected at 450 nm using a microplate reader (Biotek Instruments Inc).

### Cell apoptosis

Cell apoptosis was determined using an Annexin V/PI Apoptosis Detection kit following the manufacturer’s instructions. Cells were cultured in a 6-well plate overnight at a density of 2 × 10^5^ /well for HK2. The treated cells were collected and labeled with annexin V and propidium iodide (PI) for 15 minutes in the dark, and then apoptotic cells were analyzed via flow cytometry.

### Western blot analysis

Renal tissues or cells were lysed in a RIPA buffer with protease inhibitor. The protein concentration was measured using the bicinchoninic acid (BCA) method. Protein extracts (40 μg) were separated on SDS-PAGE gel and transferred to a PVDF membrane. The membranes were blocked with 5% skim milk at room temperature for 2 h. This was followed by overnight incubation at 4° C with monoclonal antibodies against PGC-1α (1:500 dilution, A12348; ABclonal), TFEB (1:1000 dilution, 4240S; CST), TFEB (1:500 dilution, A7311; ABclonal), P62 (1:1000 dilution, 109012; Abcam), LC3B (1:1000 dilution, 2775S; CST), Bax (1:500 dilution, A0207; ABclonal), Bcl-2 (1:1000 dilution, A11025; ABclonal), Ndufs4 (1:500 dilution, A6390; ABclonal), and ATP5b (1:500 dilution, A5769; ABclonal). The membranes were then incubated with the corresponding secondary antibodies (1:5000 dilution) at room temperature for 2 h. The immunoblots were visualized using a ChemiDoc™ imaging system (Bio-Rad, USA). Immuoblot band density was quantified via densitometry using ImageJ software. The protein expression levels were normalized against β-actin.

### Mitochondrial ROS detection

Mitochondrial ROS generation was measured with a flow cytometer or confocal microscope. For the flow cytometry analysis, cells were labeled with Mito-SOX Red (2.5 μM) for 15 min at 37° C, after which cells were washed twice with PBS and analyzed using flow cytometry. For the fluorescence microscopy analysis, after the staining of Mito-SOX Red, nuclei were stained with Hoechst (1 μg/mL) for 5 min at room temperature and washed with PBS for 3 times, the visualized images were acquired using confocal microscopy. The renal tissues removed from mice were immediately cut into 5μm thick sections, and then incubated with Mito-SOX Red in a dark and humidified container at 37° C for 15 min. After washing with PBS, digital images were captured by confocal microscopy.

### Mitochondrial membrane potential

The mitochondrial membrane potential (ΔΨm) was detected using JC-1, according to the manufacturer’s instructions. Briefly, cells were washed twice in PBS and stained with JC-1 dye (5 nM) for 30 min at 37° C. The cells were washed with PBS again and then visualized images were acquired using confocal microscopy.

### Mitochondrial mass

Cells were cultured in a 6-well plate overnight at a density of 2 × 10^5^ /well for HK2. The treated cells were collected and stained with two dyes that accumulate specifically in mitochondria at 37° C, Mito-tracker-green (50 nM, 30 min; independent of mitochondrial inner membrane potential) Mito-tracker-deep-red (100 nM, 30 min; potential-dependent mitochondrial accumulation), and analyzed the intensity of the signals by flow cytometry.

### Immunofluorescence (IF)

Cells were washed in PBS, incubated with 100 nM Mito-tracker-deep-red at room temperature for 30 min, fixed in PBS with 4% paraformaldehyde for 10 min and permeabilized with 0.1% Triton X-100 in PBS for 10 min. After locking with 1% BSA in PBS for 30 min, cells were incubated with diluted primary antibody (LC3B at a 1:200 ratio; TFEB at 1:200 ratio) overnight at 4° C, followed by secondary antibodies at 1:200 for 1 h at 37° C. After staining with DAPI for 5 min at room temperature and washing with PBS, the fluorescent signals were examined using a fluorescence microscope.

### Quantitative real-time PCR

Total RNA was extracted from cells and mouse kidney using TRIzol reagent and reverse-transcribed into cDNA using an iScript cDNA synthesis kit according to the manufacturer’s protocol. Real-time PCR was performed to assess gene expression on the CFX96 real-time PCR detection system (Bio-Rad) using SYBR Green master mix. The primers used in this study are designed by Sangon Biotech Co. Ltd. (Shanghai, China), and listed in Table 1. The relative changes in the mRNA expression of target genes were calculated using the ΔΔCt method, with GAPDH as the reference gene.

**Table 1:** Primers used for real-time PCR analysis.

**Gene**	**Primer sequence**
p62-F	CCGTCTACAGGTGAACTCCAGTCC
p62-R	AGCCAGCCGCCTTCATCAGAG
GAPDH-F	ACCACAGTCCATGCCATCAC
GAPDH-R	TCCACCACCCTGTTGCTGTA

### Measurement of cellular ATP

Intracellular ATP was measured using the ATP assay kit, as described by the manufacturer. The collected cells were lysed with lysis buffer and then centrifuged at 12000 g for 10 min at 4° C. After that, an aliquot of the supernatant plus ATP detection solution was added to a 96-well plate (96-Well Bottom Black Culture Solid Plate). Luminescence was detected using a SpectraMax M5 MultiMode Microplate Reader. ATP levels were presented as nmol/mg of protein.

### Co-immunoprecipitation

Whole-cell extracts were diluted to 1mg of protein in 100 μL of lysis buffer and precleared for 1 h at 4° C with 10 μL of protein A/G agarose beads (Beyotime Biotechnology, Jiangsu, China). After removing precleared beads via brief centrifugation, 1μg of rabbit anti-PGC-1α or rabbit anti-TFEB was added to the sample and incubated on a rocking shaker overnight at 4° C with gentle shaking, followed by addition of 20 μL of protein A/G agarose for a further 4 h incubation at 4° C. The immunoprecipitated complexes were then washed with lysis buffer three or four times and eluted from the beads with protein loading buffer. After washing, immunoprecipitates were boiled in SDS/PAGE loading buffer and subjected to western blotting analysis.

### Dual-luciferase reporter assay

The TFEB promoter reporter construct linked to luciferase (GPL3-TFEB) were provided by GenePharma (Shanghai, China). A luciferase reporter assay was performed by using a Dual Luciferase Reporter Assay Kit (DL101-01; Vazyme Biotech) as described by the manufacturer. Luciferase activity was measured at 48 h after transfection of HK2 cells with the GPL3-TFEB and GPL3-Basic constructs with the pENTER-PGC-1α expression plasmids (or pENTER empty vectors) using jetPRIME transfection reagent according to the manufacturer’s instructions. For all experiments, Renilla luciferase reporter plasmids were co-transfected into HK2 cells and used as internal controls to correct for variation in transfection efficiency among samples. Relative reporter activity to Renilla luciferase activity.

### Animals

All animal experiments were approved and conducted in accordance with the guidelines of the Institutional Animal Care and Use Committee of Sichuan University. Male C57BL/6 mice (6-8 weeks) were purchased from Dashuo Biotechnology (Chengdu, China). All animals were maintained on a 12-hour light-dark cycle in a temperature-controlled (20° C–25° C) room, fed a standard chow, and allowed free access to drinking sterile water in accordance with the Guide for the Care and Use of Laboratory Animals. After a two-week adaptive period, the mice were randomized to the following groups: normal control (NC: n=10); Zln group (n=10); Cisp group (n=10); Cisp+Zln group (n=10). Specifically, mice were intraperitoneally (i.p.) injected with Cisp (16 mg/kg, single injection) to induce AKI. For Zln treatment, Zln (15mg/kg per day, i.g.) was orally administrated for 4 days as the same time as injection of Cisp. The blood and kidneys were collected when the animals were sacrificed.

### Biochemical measurement

Serum creatinine (Crea) and blood urea nitrogen (BUN) in the mice were detected by an auto-analyzer (Cobas 6000, Roche Diagnostics, Switzerland) using commercial kits.

### Histological examination

The kidney tissues were dissected and fixed in 4% paraformaldehyde, embedded in paraffin, sliced into 5 μm sections, stained with hematoxylin-eosin (HE) and periodic acid-Schiff (PAS), and observed by light microscopy. For immunohistochemistry (IHC) staining, kidney sections were deparaffinized in xylene and rehydrated in graded ethanol concentrations and antigen retried with a citrate buffer. After the inactivation of endogenous peroxidase with 3% H2O2, sections were locked with 1% BSA in PBS for 30 min and incubated with diluted primary antibodies (Kim-1 and PGC-1α) overnight at 4° C. After washing with PBS, sections were stained with horse radish peroxide (HRP)-conjugated secondary antibodies. Images of stained sections were captured by light microscopy.

### Renal tissue TUNEL staining

The apoptotic cells in frozen renal sections were assessed using TUNEL staining (Promega, Madison, WI, USA) according to the manufacturer’s instructions. The cell nuclei were stained with DAPI; positive staining with nuclear DNA fragmentation was detected by fluorescence microscopy.

### Transmission electron microscopy (TEM)

Fresh mouse renal tissues were fixed in 2.5% glutaraldehyde at pH 7.43, dehydrated, and embedded in Epon. The embedded tissues were cut into ultrathin sections, stained with 5% uranyl acetate and lead citrate, and analyzed by TEM.

### Statistical analysis

Experiments were performed at least three times and quantitative data are expressed as mean ± S.E.M. Student’s two tailed unpaired *t* test for two groups or one way ANOVA (Tukey test) for multiple groups was used to detect statistical significance (defined as *p* < 0.05). GraphPad Prism 6.0 was used for the statistical analysis.

## Supplementary Material

Supplementary Figures
