# Supplementary material for: PGC-1α alleviates mitochondrial dysfunction via TFEB-mediated autophagy in cisplatin-induced acute kidney injury
Source: Aging (Albany NY). 2021 Mar 10;13(6):8421–39. doi: 10.18632/aging.202653 (PMC8034953; doi:10.18632/aging.202653)
Supplement: Supplementary Figures [file aging-13-202653-s001.pdf]

SUPPLEMENTARY FIGURES

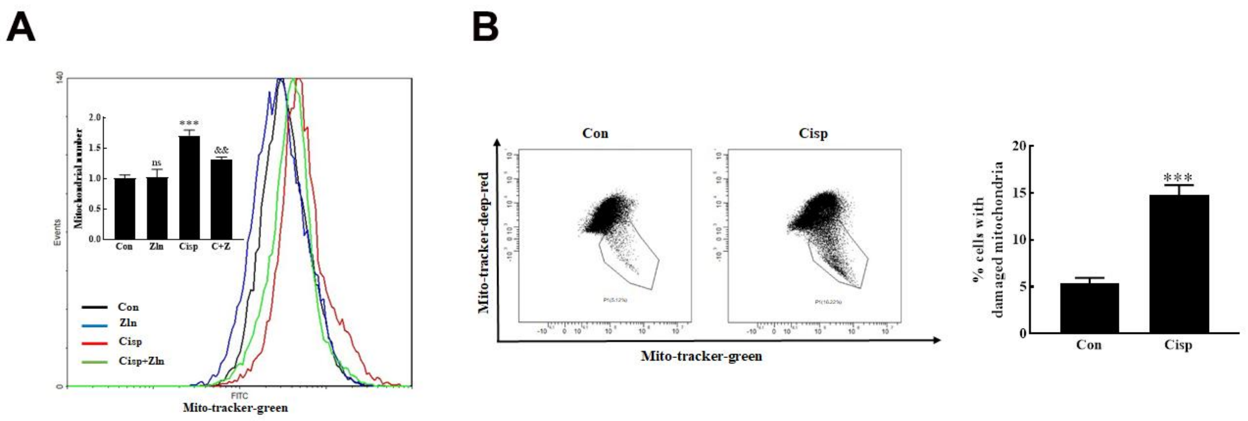

Supplementary Figure 1. Mitochondrial dysfunction induced by cisplatin caused the increase of mitochondrial mass. (**A**) Mitochondrial number was assessed by flow cytometry with Mito-tracker-green staining (50 nM, 30 min). (**B**) Co-staining of Mito-tracker-green (50 nM) and Mito-tracker-deep-red (100 nM) dyes in Cisp-treated HK2 cells showing increased population of dysfunction mitochondria (Mito-tracker-greenhi Mito-tracker-deep-redlow population (P1) by flow cytometry). Data are provided as the mean ± SEM, n=3 independent experiments. *P < 0.05, **P < 0.01, ***P < 0.001 vs. Con; &P < 0.05, &&P < 0.01 vs. Cisp. (Con, control; Zln, ZLN005; Cisp, cisplatin; C+Z, cisplatin + ZLN005).

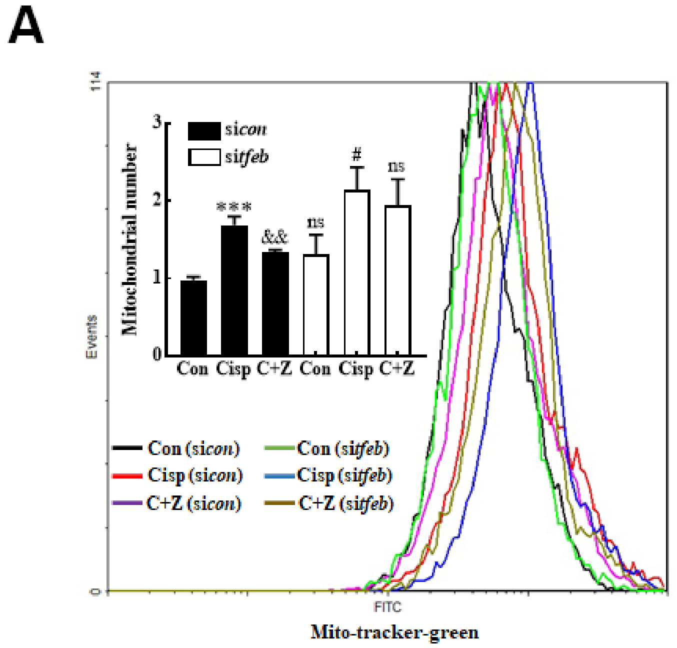

Supplementary Figure 2. The effect of sitfeb on mitochondrial number. (**A**) HK2 cells were transfected with control siRNA (sicon, black column) or TFEB siRNA (sitfeb, white column) for 6 h and treated with cisplatin in the presence or absence of ZLN005 for 48 h. Mitochondrial number was assessed by flow cytometry with Mito-tracker-green staining. Data are provided as the mean ± SEM, n=3 independent experiments. *P < 0.05, **P < 0.01, ***P < 0.001 vs. Con; &P < 0.05, &&P < 0.01 vs. Cisp; #P < 0.05 vs. sitfeb. (Con, control; Zln, ZLN005; Cisp, cisplatin; C+Z, cisplatin + ZLN005).
